# A mechanism for semaphorin-induced apoptosis: DNA damage of endothelial and myogenic cells in primary cultures from skeletal muscle

**DOI:** 10.18632/oncotarget.25200

**Published:** 2018-04-27

**Authors:** Haynes Shek Hei Yuan, Sachin Katyal, Judy E. Anderson

**Affiliations:** ^1^ Department of Biological Sciences, CancerCare Manitoba, Winnipeg, MB, Canada; ^2^ Department of Pharmacology and Therapeutics, CancerCare Manitoba, Winnipeg, MB, Canada; ^3^ University of Manitoba, Research Institute in Oncology and Hematology, CancerCare Manitoba, Winnipeg, MB, Canada

**Keywords:** DNA damage, semaphorins, apoptosis, endothelial cell, angiogenesis

## Abstract

One hallmark of cancer is its ability to recruit a vascular supply to support rapid growth. Suppression of angiogenesis holds potential as a second-line or adjuvant therapy to stunt cancer growth, progression, metastasis, and post-resection regeneration. To begin to test the hypothesis that semaphorin 3A and 3F together, will induce endothelial cell apoptosis by inducing DNA damage, mixed primary cultures isolated from normal adult mouse skeletal muscle were treated for 48 hr with Sema3A ± Sema3F (100ng/mL). Changes in surviving-cell density, DNA synthesis, DNA repair (gamma-Histone 2AX, γH2AX, an indirect measure for DNA damage), and apoptotic DNA fragmentation (TUNEL staining) were assayed in cultures of CD31+ endothelial and desmin+ muscle cells. Sema3F increased DNA damage-associated DNA repair in both cell types. Co-treatment with Sema3A+3F increased γH2AX staining ~25-fold over control levels, and further increased apoptosis compared to control and Sema3A alone. Results were negated by treatment with neutralizing anti-semaphorin antibodies and are interpreted as suggesting that Sema3A may sensitize endothelial but not muscle cells to Sema3F-induced DNA damage. These preliminary findings on a complex system of interacting cells may contribute to developing applications that could target angiogenic regulatory mechanisms for their therapeutic potential against cancer progression and metastasis.

## INTRODUCTION

Class-3 semaphorins (Sema3s) are multifunctional secretory proteins; their secretion provides them capacity to exert autocrine and paracrine effects in many signaling pathways [1]. While the axon-guidance properties of Sema3 proteins are well recognized [2–5], more recently, Sema3A was implicated in regeneration of skeletal muscle tissue after injury [6–12] and re-establishment of motor neurite contact at neuromuscular junctions on newly formed muscle fibers (reviewed in [13]).

Sema3 regulation of nervous and vascular systems displays parallel processes [13, 14]. Modern research is establishing the potent role of semaphorins in tissue angiogenesis, as our understanding of complex cell-signaling pathways is dissected. For example, Sema3A prevents endothelial cell survival and mobility [15, 16], and interacts with cells in the immune system [17, 18]. In combination with Sema3F secretion, cultured endothelial cells engineered to secrete both Sema3F and Sema3A were induced into apoptosis [19]. However, while class-3 semaphorins are well-established as angiogenic regulators in many systems including the musculoskeletal system and some cancerous tumors, the molecular basis of their regulatory effects on vascular sprouting and elongation is not clear. Since angiogenesis is crucial to growth and repair of normal tissues and also supports cancer growth and metastasis, a better understanding of the molecular processes that underpin regulation of angiogenesis will help elucidate new cancer-suppressing therapies [20].

Experiments were designed to explore the mechanisms underlying previously reported endothelial cell apoptosis induced by semaphorins, and to begin to test the hypothesis that endothelial cell apoptosis induced by combined treatment with Sema3A+3F occurs through DNA damage. Primary mixed cultures isolated from mouse skeletal muscle tissue were used in these experiments since they contain vascular endothelial cells, the primary cell of interest in this study, and a representative of somatic cells, myogenic precursors derived from muscle stem cells (satellite cells) in addition to other cells. Endothelial and myogenic cells are both targeted by class-3 semaphorins [13]. Ideally, a treatment directed to killing vascular tissue would not affect normal somatic cells, so the use of primary mixed cultures from muscle allowed detection of differential treatment responses between the two cell types.

Primary mixed cultures containing mainly endothelial and myogenic cells were treated with semaphorins and treatment effects were assayed using immunostaining approaches. Since Ser-139 phosphorylation of histone protein H2AX into γ-H2AX is an important early step in the DNA-damage repair process, the appearance of γ-H2AX foci was used as an indirect reporter of nuclear DNA damage [21]. TUNEL staining was used to identify the prevalence of apoptosis associated with this DNA damage in cells in the mixed cultures. Inhibitor studies using neutralizing antibodies to Sema3A ± Sema3F were used to confirm that differences from control cultures were related to the two semaphorins [22]. The differential effects of single and combined treatment on endothelial vs. myogenic cells that induced DNA synthesis, damage-associated DNA repair and apoptosis, were abrogated by neutralizing antibodies, and suggested the possibility that Sema3A sensitizes primary endothelial cells to anti-angiogenic effects of Sema3F.

## RESULTS

**Figure 1:**
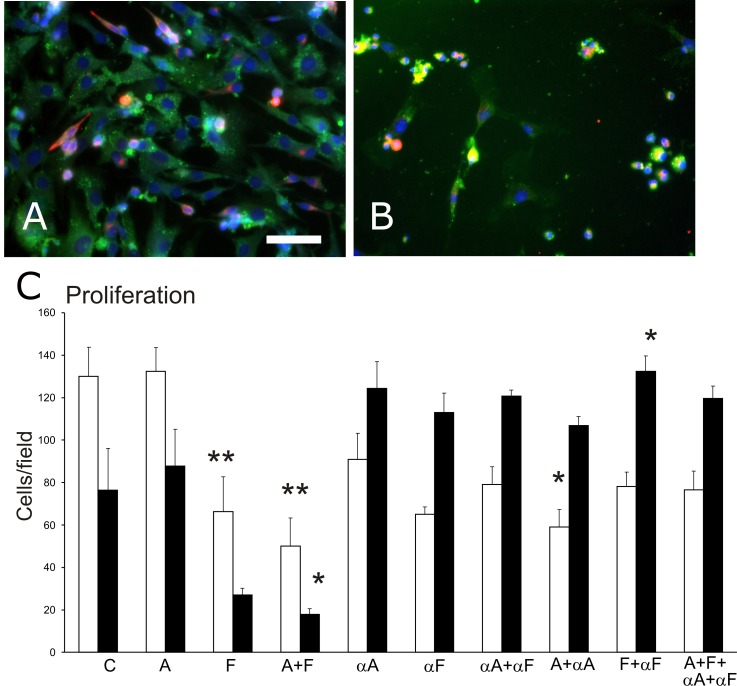
Cell density in primary muscle-derived cell cultures surviving after 48-hr treatments in culture Cultures were immunostained to identify myogenic desmin+ cells (fluorescent red) and CD31+ endothelial cells (fluorescent green), and nuclei were counterstained with DAPI (blue). **(A)** a representative control culture. **(B)** a culture treated with Sema3A and Sema3F that displays fewer cells than in the control group. Bar=50μm. **(C)** a graph of changes in the number of surviving cells per field (mean, SEM) for endothelial cells (white bars) and myogenic cells (black bars) after cultures were treated for 48 hr with 100ng/mL of one or both of Sema3A (A) and Sema3F (F); one or both of anti-Sema3A (αA) and anti-Sema3F (αF) antibodies; or a combination of semaphorin(s) and antibodies, compared to control untreated cultures (C). Asterisks indicate significant difference from control group (Tukey's test): ^*^p<0.05; ^**^p<0.01.

Cells in the mixed cultures were identified by immunostaining as endothelial or myogenic (Figure 1A, 1B). The ratio of endothelial-to-myogenic cells did not vary among control cultures from different cell isolations. The ratio of endothelial to myogenic cells was 1.78 ± 0.26 in control cultures after the 48-hr experimental period; the ratio was significantly reduced by treatment with Sema3A (to 0.68 ± 0.09). The ratio of endothelial-to-myogenic cells in Sema3F cultures (1.03 ± 0.02) was not different from that in control cultures. The density of cells surviving the experimental period (Figure 1C) was significantly different among the treatment groups (ANOVA, p<0.01), and the effect of treatment varied between cell type for the two semaphorins (ANOVA, p<0.01). Specifically, Sema3F treatment reduced the density of endothelial cells (p<0.05), while Sema3A treatment did not affect the density of either type of cell at the end of the 48-hour treatment period compared to control. Combined treatment with Sema3A+3F significantly reduced the cell density for both endothelial (p<0.01) and muscle cells (p<0.05).

In cultures treated with antibodies to bind to and thereby neutralize the effects of semaphorin(s) (Figure 1C), cell density closer to that of controls, and in 5 of the 6 antibody-treated groups did not differ from control levels. Results also show that treatment with antibodies alone, affected cell density compared to control (ANOVA, p<0.05), likely due to endogenous release of semaphorins from the isolated primary cells into the culture medium.

### DNA synthesis

**Figure 2:**
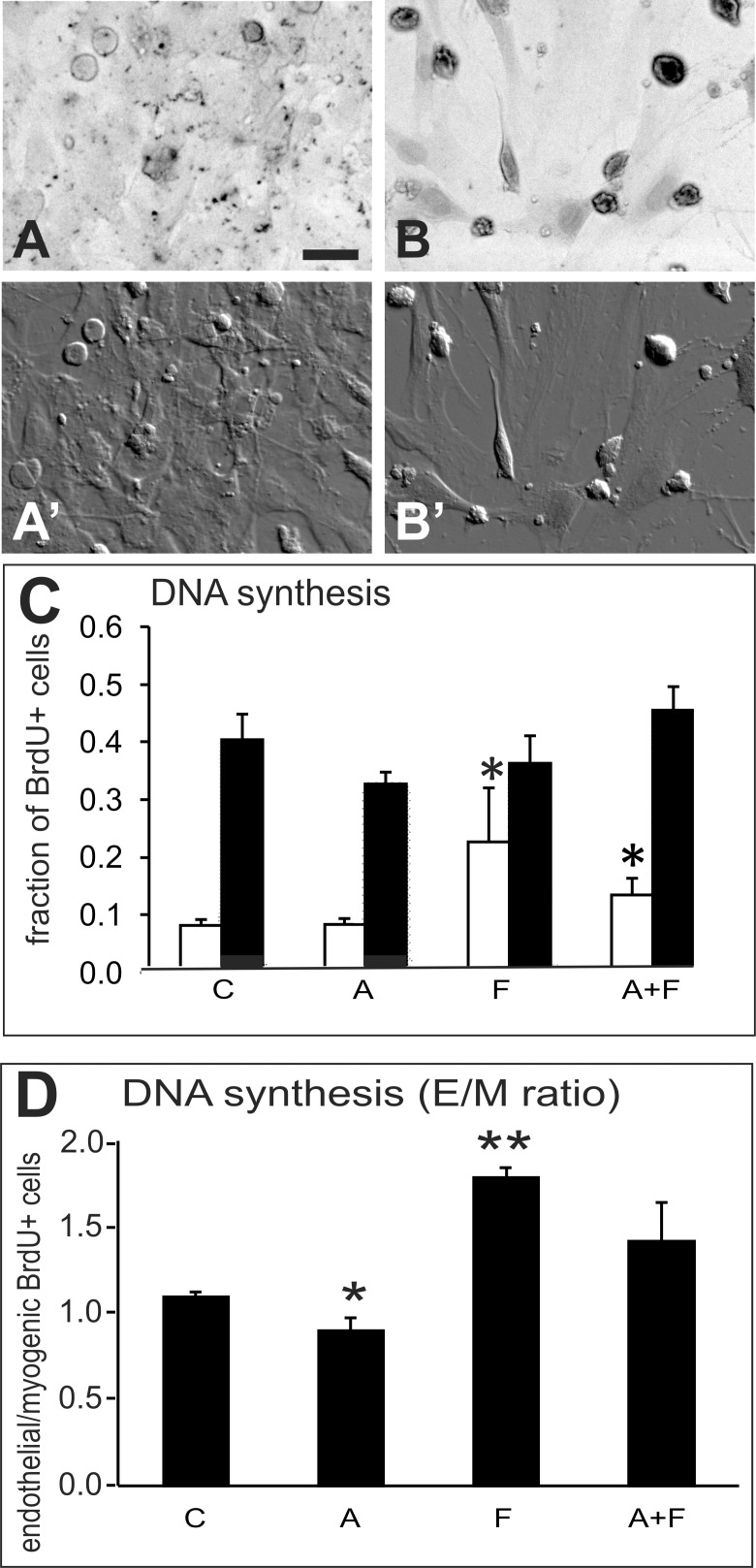
DNA synthesis experiment Analysis of DNA synthesis in nuclei of primary muscle-derived cell cultures after BrdU incorporation into DNA, detected by non-fluorescent HRP-DAB staining in combination with immunofluorescent detection of endothelial (CD31+) and myogenic (desmin+) cells. Cultures were exposed to BrdU for 1hr before fixation and immunostaining. Panel **(A)** a control culture in which only a few cells contain lightly stained nuclei. **(B)** a culture treated with combined Sema3A+3F showing cells with darkly stained BrdU+ nuclei. **(A’ and B’)** are DIC images of the same fields as in A and B. Bar=25μm. **(C)** graph of the proportion of BrdU+ endothelial cells (white bars) and myogenic cells (black bars) in control cultures without treatment, and cultures treated for 48hr with 100ng/mL Sema3A (A), Sema3F (F) or both proteins (A+F). **(D)** graph of the ratio (E/M ratio) of BrdU+ endothelial cells to BrdU+ myogenic cells from cultures in the same experiment as in C. Treatments with Sema3F alone (F) or Sema3F combined with Sema3A (A+F), significantly increased DNA synthesis in endothelial cells. Asterisks indicate significant difference from control group (Tukey's test): ^*^p<0.05; ^**^p<0.01.

A DNA-synthesis assay was used as a second approach to counting cells in the proliferation assay. The thymidine analogue, bromodeoxyuridine (BrdU), which is incorporated into DNA during DNA synthesis, was detected using non-fluorescent 3,3’-diamino-benzidine (DAB) staining (Figure 2A, 2B) together with fluorescent immunostaining to detect cell type. The proportion of BrdU+ nuclei was determined for each of the endothelial cells and muscle cells in each culture dish. This assay revealed that DNA synthesis increased significantly in cultures treated with Sema3F (p < 0.05), either alone or in combination with Sema3A (Figure 2C). The fraction of BrdU+ cells differed between endothelial and muscle cells in control cultures as shown by a plot of the ratio of BrdU+ endothelial cells to BrdU+ myogenic cells (ANOVA, p<0.001) and was reduced after Sema3A treatment and increased after Sema3F treatment (Figure 2D).

### DNA damage

**Figure 3:**
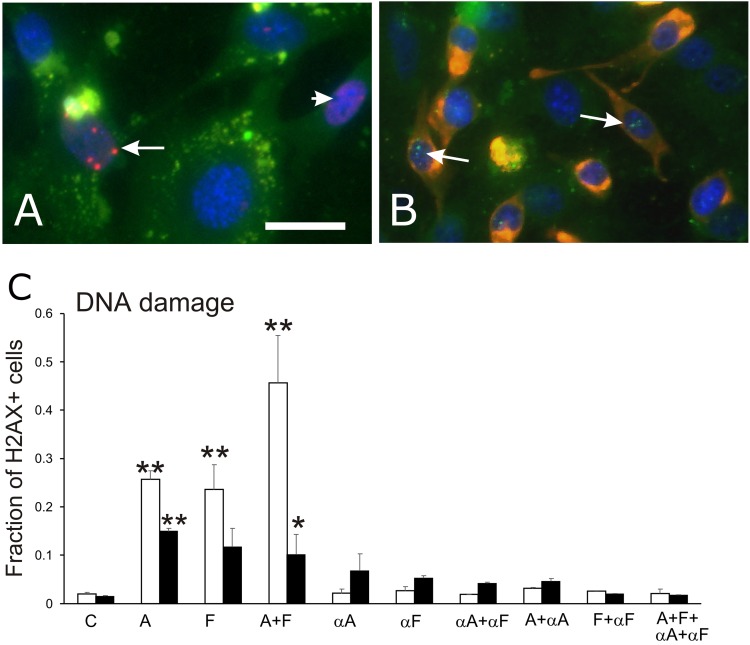
γH2AX assay for DNA repair associated with DNA damage Anti-γH2AX immunofluorescence was used to detect repair of DNA strand breaks in primary muscle-derived cultures. **(A)** immunofluorescence image of γH2AX+ foci (fluorescent red dots indicated by arrow) in the nucleus of a green fluorescent CD31+ endothelial cell. A nucleus with minor DNA damage identified by small patches of red fluorescence, is indicated by the arrowhead. **(B)** image of γH2AX+ foci of DNA damage (fluorescent green dots, indicated by arrows) in the nuclei of two fluorescent red desmin+ myogenic cell. Bar=10 μm. **(C)** graph of the proportion (mean, SEM) of nuclei with γH2AX+ foci in endothelial cells (white bars) and myogenic cells (black bars) in control cultures (C), cultures treated with Sema3A (A), Sema3F (F) or both proteins (A+F), and cultures treated with one or more neutralizing antibodies for Sema3A (αA) and Sema3F (αF), with or without exposure to semaphorins 3A and/or 3F. Asterisks indicate significant difference from control group (Tukey's test): ^*^p<0.05; ^**^p<0.01.

Phosphorylation of H2AX protein on Ser-139 (γH2AX) was used as an indicator of DNA damage, and was detected separately in endothelial and myogenic cells (Figure 3A, 3B). Results of the γH2AX assay (Figure 3C) showed that semaphorin treatments induced DNA damage in the cultured cells (ANOVA, p<0.005). The extent of DNA damage also differed between endothelial and muscle cells (ANOVA, p<0.005). All semaphorin treatments significantly increased DNA damage in endothelial cells compared to controls (p<0.01 for Sema3A and Sema3F; p<0.05 for combined Sema3A+3F). Application of semaphorin-neutralizing antibodies completely eliminated the effects of semaphorin treatments as γH2AX-marked levels of DNA damage were equivalent to those of controls.

### Apoptosis (TUNEL staining)

**Figure 4:**
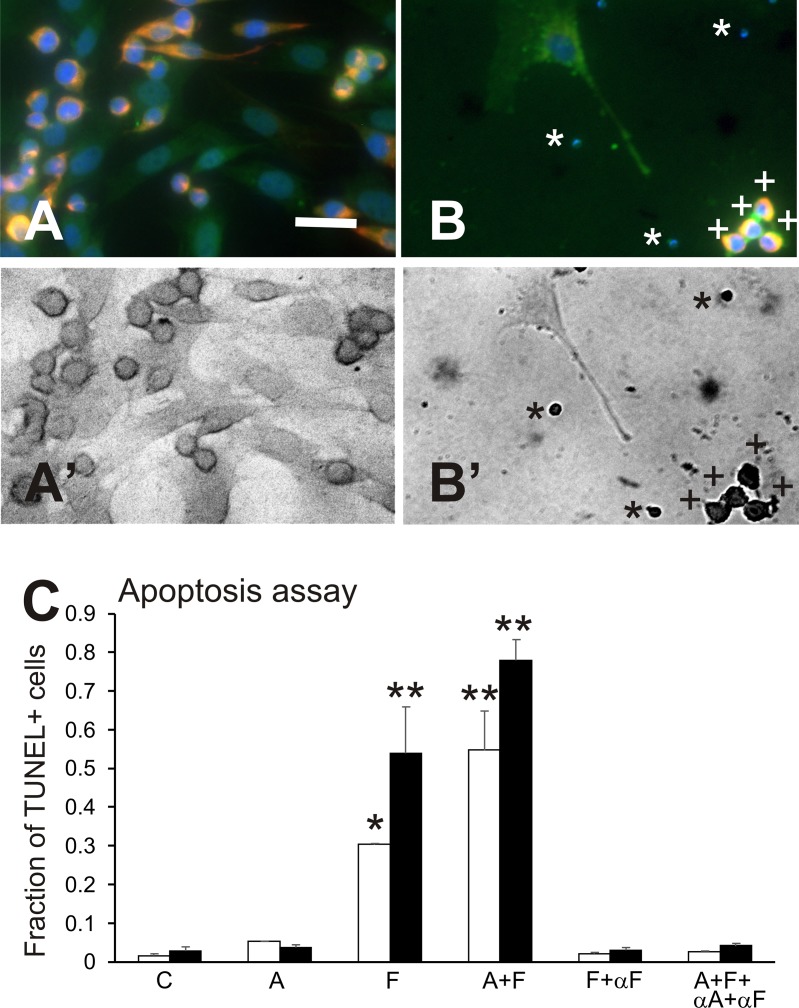
Apoptosis assay Results of experiments on apoptosis, using an assay combining immunofluorescence and TUNEL staining. **(A and A’)** a control culture visualized in fluorescence (A) and in the bright-field view showing absence of dark nuclei after TUNEL staining (B’); elongated muscle cells (red fluorescence) have slightly darker staining in their nuclei than the paler endothelial cells in brightfield due to differences in cell thickness. **(B and B’)**, a culture that received combined treatment with Sema3A+3F, showing an endothelial cell with an extension near a group of small, multi-colour fluorescent bodies (A) that are darkly stained and TUNEL+ nuclei in B’ (indicated by “+”). There are also small TUNEL+ apoptotic bodies in B’ (indicated by “^*^”) that in B, are stained by DAPI+ for DNA. Bar=20μm. **(C)** graph of results for the late-apoptosis assay showing the fraction of cells that were TUNEL+, indicating the presence of DNA fragmentation, in groups treated with 100ng/mL of Sema3A (A), Sema3F (F) or both semaphorins (A+F); Sema3F plus neutralizing antibody to Sema3F (F+αF), or both semaphorins plus antibodies to both epitopes (A+F+ αA+αF), compared to control cultures. Any treatment that included Sema3F resulted in an increase in the fraction of TUNEL+ endothelial cells (white bars) and myogenic cells (black bars). Neutralizing αF antibodies to Sema3F restored the fraction of cells showing DNA fragmentation to control levels. Sema3A alone did not change the fraction of cells demonstrating DNA fragmentation, as detected with TUNEL staining. Asterisks indicate significant difference from control group (Tukey's test): ^*^p<0.05; ^**^p< 0.01.

DNA fragmentation, a hallmark for cell apoptosis, was identified using TUNEL staining in endothelial or myogenic cells (Figure 4A, 4B). Results of the TUNEL assay (Figure 4C) showed overall, that treatment increased the proportion of cells with DNA fragmentation in primary cultures compared to control cultures (ANOVA, p<<0.001). While Sema3A alone did not change the proportion of TUNEL+ cells, Sema3F increased the level of apoptosis in both endothelial cells and muscle cells by 18-fold (p<0.05 and p<0.01, respectively). Combined Sema3A+3F treatment also induced 33- and 27-fold increases in apoptosis over control cells in endothelial and muscle cells, respectively (p<0.01 for both comparisons).

In parallel cultures from the same cell preparation, anti-semaphorin antibodies completely reversed the appearance of apoptosis, as indicated by TUNEL staining (Figure 4C). The fraction of TUNEL+ cells in cultures treated with neutralizing antibodies to Sema3A, Sema3F, and against both Sema3A and Sema3F, did not differ from the respective cell-type control cultures.

## DISCUSSION

Primary mixed cultures from skeletal muscle were used as a preliminary test of the hypothesis that combined treatment with Sema3A+3F induces cell apoptosis to vessel endothelial cells as a result of DNA damage. Results suggest that anti-angiogenic properties of class-3 semaphorins may be related to, or at least include the possibility of DNA damage that is induced by semaphorins, based on an assay of γH2AX localization, an indirect indicator of DNA repair that would ensue from damage. Accumulation of DNA damage would lead to cell apoptosis, which was assayed with TUNEL staining in this study. The co-application of antibodies to the two semaphorins either completely or partially neutralized semaphorin effects on γH2AX staining and the TUNEL assay.. Findings provide preliminary new insight into the possible role of class-3 semaphorins to regulate angiogenesis in normal tissue, modeled as mixed primary cells from skeletal muscle, and thus suggest their potential to serve as a cancer-suppressive second-line treatment to inhibit tumour growth and propagation. The importance of finding anti-cancer treatments suggests the findings from primary-cell cultures merit further investigation with more specific probes of DNA damage and mechanisms of DNA repair and apoptosis in tracking the cell-by-cell multi-variate responses to semaphorins 3A and 3F [23–25].

The study of cell density in mixed primary cultures showed that Sema3A and Sema3F both had effects on cell survival. Sema3F reduced the density of cells surviving the culture period and combined treatment with Sema3A+3F further reduced cell density. Interestingly, the reduction in cell density was accompanied by an increase in DNA synthesis, measured by 1-hr exposure to BrdU, after semaphorin exposure, particularly to combined Sema3A+3F. These findings cannot be fully explained by a DNA-repair response during TUNEL+ apoptosis, as assayed by γH2AX immunostaining, an indirect probe for DNA damage. Mechanisms of DNA repair such as nucleotide-excision, homologous recombination and non-homologous end-joining [26, 27] all require DNA synthesis [28] and would be labelled by BrdU incorporation. While cell-cycle duration may vary between different types of cells, additional assays of proliferation (e.g., Ki67 or flow-cytometry studies using BrdU and propidium iodide [29]) and metabolic activity (e.g., cell reduction of tetrazolium salts such as MTT and WST1 [30, 31]) would complement findings on surviving-cell density and further account for BrdU labeling of DNA synthesis in relation to cell cycling and/or DNA repair during apoptosis.

Interactions between myogenic and endothelial cells in the primary mixed cultures isolated from skeletal muscle are likely implicated in the results of these preliminary experiments. For example, myogenic cells are known to secrete Sema3A [9, 10, 12, 13, 32], and effects of exposure to antibodies raised against Sema3A and Sema3F by otherwise untreated cultures indicated the possibility of differential semaphorin signaling between the endothelial and myogenic cells. Cells of other origin were also present, likely including fibroblasts, tissue-resident macrophages, and Schwann cells in proportions that would among muscles. The collection of tissue from many muscles standardized the relative proportions of desmin+ and CD31+ cells across preparations, although cell-by-cell tracking of responses with multiple concurrent assays would be valuable for exploring cell-type-specific responses over time in the mixed cultures [25]. While it is not known whether effects of Sema3A and/or Sema3F treatment would be the same if mixed primary cultures were exposed at different times post-plating, studies of various cell types over a range of known proportions using cell lines for example, could reveal the primary source and effector type(s) of cell involved in treatment responses.

The γH2AX immunostaining experiments combined with the TUNEL-staining assay for apoptosis suggest that the observed cell death is associated with repair of DNA, possibly from DNA damage in endothelial and muscle cells. While this approach could not conclusively resolve the findings of reduced cell density and increased DNA synthesis in Sema3F-treated groups, distinct γH2AX+ foci marked repair of DNA strand breaks t in endothelial and myogenic cells after Sema3F treatment. There was a negative relationship between the proportion of cells with nuclear γH2AX and overall density, which is consistent with the hypothesis that Sema3A+3F together will induce apoptosis via DNA damage in endothelial cells but does not provide definitive proof. However, it is interesting to speculate that finding more γH2AX+ foci after combined Sema3A+3F treatment than after either semaphorin alone, there is a possibility that Sema3A sensitized endothelial cells in particular, to DNA damage induced by Sema3F. Possible synergy between semaphorins 3A and 3F in inducing apoptosis, could be useful in developing treatments to restrict angiogenesis while minimizing impact on other types of cells.

TUNEL staining is one of many assays for apoptotic events resulting from DNA damage. Results of the assay for DNA fragmentation, which occurs late in the process of apoptosis, showed induction of fragmentation and apoptotic bodies in both endothelial and muscle cells in the mixed primary cultures. The proportion of cells undergoing apoptosis was higher after Sema3F and Sema3A+3F treatments than in control cultures. Staining with 4,6-diamidino-2-phenylindole (DAPI) confirmed DNA fragmentation during cell-death events during Sema3F treatment. Use of a microculture-kinetic (MiCK) approach to quantify apoptosis [33] could reveal if effects of Sema3A+3F synergize with other anti-cancer therapies.

The use of antibodies to neutralize semaphorin activity in culture restored the cell density and the γH2AX-associated DNA-repair activity as well as the level of apoptosis to levels comparable to control. The degree of restoration by anti-semaphorin antibodies varied among the different assays. Antibodies completely restored the low levels of DNA repair and apoptosis seen in control cultures, compared to increased γH2AX-associated DNA repair after exposure to one or both semaphorins. By comparison, anti-semaphorin antibodies only partly reversed the decline in surviving-cell density, an effect that was greater for myogenic than endothelial cells in the mixed primary cultures. Since semaphorin antibodies in 40-fold excess of respective epitopes did not completely neutralize semaphorin effects, it would be useful to conduct semaphorin-knockdown experiments in one or more cell-types in a mixed culture and compare those results to effects on cells from conditional-knockout transgenic animals [11] to confirm the impact of semaphorins on proliferation, damage-associated DNA repair, and apoptosis.

Interestingly, vascular pericytes and myogenic cells both express c-met receptor which binds the ligand, hepatocyte growth factor (HGF) [34]. HGF is produced by proliferating myogenic cells (and anti-inflammatory macrophages [8]), and is a key signaling molecule in the pathway that activates metabolically and mitotically quiescent muscle satellite cells to cycle and migrate [35, 36]. HGF from cultured myogenic cells may have influenced the differential responses by endothelial and myogenic cells to Sema3A and 3F, as found in studies of human colon cancer in a mouse transgenic strain [37]. Amplification of MET expression in some cancers [38] would affect interaction of HGF with proteins in the senescence-associated secretory phenotype [39], as would the stage of angiogenesis in the growth, carcinogenesis and metastasis of tumors in response to Sema3A signaling [40, 41]. The role of the mammalian target of rapamycin (mTOR) complex in autocrine effects of Sema3A that promote cancer formation [42] and help regulate disposal of senescent cells with DNA damage [39, 43] remains to be explored. The present experiments on cells derived from a highly stable vascular network in wildtype adult skeletal muscle were used to model early aspects of angiogenic regulation and results, particularly the apparently selective, seemingly sensitizing effect of Sema3A on endothelial cell responses to Sema3F. This relates to an apparent inconsistency in the findings, since Sema 3A did not affect apoptosis in myogenic or endothelial cells (Figure 4C), DNA synthesis of either cell type (except as a ratio of BrdU+ cells in Figure 2D), or cell density, yet still seemed to induce a dramatic augmentation in DNA damage in both endothelial and myogenic cells (Figure 3C). It is possible that the γH2AX-detected DNA repair found after Sema3A treatment, was able to compensate for DNA damage without affecting cell survival or DNA synthesis, or inducing apoptosis. A high level of DNA repair via γH2AX and other mechanisms [24] during Sema3A exposure may make cells more sensitive to effects of Sema3F. There may also be a “threshold effect” wherein only the combination of both semaphorins affects surviving cell number, as described in our previous DNA-damage publication [44]. Further, Sema3A and 3F may induce different classes of DNA damage, which may affect the time course, magnitude, and differential impact on myogenic and endothelial cells, of the final apoptotic signal.

The differential responses to DNA damage and apoptosis are intriguing, as results demonstrate distinctive responses specific to different cell types, and to single or co-treatment with Sema3A and Sema3F. First, there was a differential in responses to either Sema3A or Sema3F exposure by endothelial vs. myogenic cells, while the combined treatment with two semaphorins together, resulted in “co-operative” DNA damage in both endothelial and myogenic cells; this was accompanied by substantial cell apoptosis in myogenic cells and a more moderated effect in endothelial cells. However, literature shows that cell-type differences in response are not surprising, considering the well-documented differential DNA damage/repair effects amongst semaphorins, and reported differences in responses to such damage between these two cell types. For example, Sema3F expression enhances cellular sensitivity to DNA damage and promotes pro-apoptotic factors [45], effects that were not associated with Sema3A. Similarly, Sema3F expression in endothelial cells also induced apoptosis while expression in vascular and lymphatic endothelial cells provided anti-tumour properties [46]. Interestingly, Sema3F expression is also associated with reduced progression of lung and colorectal cancers, suggesting it has unique contributions to cellular genomic stability [47, 48]. Consistent with our study, these reports support the notion that Sema3F has unique properties that sensitize cells to DNA damage-induced apoptosis. As well, as measured by γH2AX, endothelial cells have specific resistance to DNA damage compared to other cell types [49], and the induction of DNA damage resulted in increased endothelial cell proliferation and neovascularization [50], indicating that particular levels of DNA damage may promote endothelial cell regeneration and withstand higher thresholds of damage. Together, these studies indicate that endothelial cells have unique properties that render them more resistant to higher levels of DNA damage-induced apoptosis than other types of cell. In contrast, myogenic cells are have an inherent deficiency in DNA repair [51], likely via reduced expression of strand-break repair factors [52] that render them more sensitive to lower levels of DNA damage. Therefore, the intriguing differential effects of DNA damage and apoptosis between endothelial and myogenic cells, are likely attributable to unique cell-type specific thresholds for DNA damage that result in cell death.

Overall, results demonstrated that Sema3F induced apoptosis accompanied by reduced cell survival and increased damage-associated DNA repair in both endothelial and myogenic cells in mixed primary cultures from skeletal muscle. Sema3F was more potent in the presence of co-treatment with Sema3A, especially in endothelial cells. Mixed cultures from skeletal muscle were used as an *in vitro* approach to modeling a tumor-resistant tissue [53] with highly stable, metabolically responsive endothelial cells. Semaphorin effects may be higher for highly proliferative tumor-derived endothelial cells. These preliminary findings encourage future research into the potential of semaphorins, particularly the combination of Sema3A+3F, in second-line cancer-suppressive treatments, to target endothelial cells and slow or restrict tumor growth.

## MATERIALS AND METHODS

### Cell culture

Primary cells isolated from mouse skeletal muscle were used as the model system for this study, as approved by the institutional Animal Protocol Review Committee (F16-031). Skeletal muscles including thoracic diaphragm were dissected from mice according to established protocols [54] with slight modification. Muscle tissue was placed into Hank's Balanced Salt Solution (Sigma-Aldrich, Oakville, ON, Canada) and chopped into a fine slurry with a sterile razor blade. The slurry was digested for 3.5 hours in a solution containing 1mg/mL of each of collagenase and dispase/collagenase (Sigma-Aldrich). Enzyme activity was quenched with Dulbecco's Minimum Essential Medium (Sigma-Aldrich) containing 20% horse serum (Invitrogen). The suspension was filtered through sterile 40 μm mesh to remove tissue debris and centrifuged for 10 mins at 1500 rcf (Baxter Megafuge 1.0R), washed with HBSS, and centrifuged again. The pellet was re-suspended in medium (DMEM plus 20% HS and antibiotic/antimycotic) and plated on coverslips pre-coated with 0.2 % weight/volume gelatin placed in 35 mm Petri dishes (ThermoFisher Scientific, Burlington, ON, Canada).

Cultures were maintained at 37°C in 5% CO_2_ for 140 hr (70% confluence) before treatment. This low level of confluence was selected to prevent the fusion of myoblasts into myotubes which occurs in higher density differentiating cultures. Medium containing one of 3 treatments was added to each culture for 48 hours: 100ng/mL of Sema3A, 100ng/mL of Sema3F or 100ng/mL of each of Sema3A+Sema3F [55]. Control dishes received medium alone. In each experiment, there were 3-8 dishes per treatment group.

Each experiment utilized independent preparations of cells isolated and pooled from muscle tissues dissected from n=4-6 mice. The treatment groups reported in each figure were conducted cultures plated in a single experiment on the same cell preparation.

### Immunostaining

After 48 hr, cultures were fixed in 4% paraformaldehyde in phosphate-buffered saline (PBS) at room temperature for 10 mins and rinsed in PBS. This time-period was selected since Sema3A is made by myoblasts in early differentiation [12] and siRNA knockdown of Sema3A in culture affects expression of muscle regulatory genes and myosin isoforms within 24-48 hr [11]. Cells were immediately immunostained using primary and secondary antibodies following the IHCWorld protocol [56] to detect myogenic cells (rabbit anti-desmin (1:100) and secondary goat anti-rabbit IgG (1:200) conjugated with Alexa Fluor-594, Abcam, Toronto, ON, Canada) and endothelial cells (mouse anti-CD31 (1:100) and secondary goat anti-mouse IgG (1:200) conjugated with FITC, Abcam). Cells were counterstained with 4,6-diamidino-2-phenylindole (DAPI) using a 1:10000 dilution of a 1mg/mL stock solution [12], and coverslips were mounted with Vectashield onto cleaned glass slides and allowed to dry.

Counts of immunostained CD31+ and desmin+ cells in culture dishes were used to assess the effects of different treatments on the density of the surviving cell populations. The total number of desmin+ myogenic cells and CD31+ endothelial cells per field were counted from images captured at 200X from 8 non-overlapping fields per coverslip, stained as described below. This assay for cell type was performed simultaneously with other assays for DNA synthesis, DNA damage, or TUNEL staining.

### DNA synthesis

The rate of DNA synthesis was assayed by adding 30 μL/mL of a 10mg/mL stock solution of bromodeoxyuridine (BrdU) to cultures, 1 hour before fixation. BrdU uptake was assayed by non-fluorescent IHC in combination with fluorescent IHC for CD31+ and desmin+ cells using rat anti-BrdU primary antibody (1:100, Abcam) and secondary goat anti-rat IgG conjugated to horseradish peroxidase (1:200, Abcam), and detected with 1mg/mL 3,3’-diamino-benzidine (DAB, Sigma-Aldrich) and 0.02% hydrogen peroxide (Sigma-Aldrich) in PBS [32, 57, 58]. The BrdU+ proportions of desmin+ and CD31+ cells were calculated from counting all cells in photographs of 8 non-overlapping fields (200X) per dish (see below).

### DNA damage

To quantify DNA damage, fixed cultures were immunostained for γH2AX, a well-known marker for DNA strand breakage localized at the site of DNA repair in early-stage apoptosis [59–61]. Foci containing γH2AX were detected in desmin+ cells (red fluorescent by IHC) using Alexa Fluor 488 conjugated anti-γH2AX Phospho (ser139) antibody (1:100, made in mouse, BioLegend, Aachen, Germany) and visualized as green fluorescence. Similarly, for CD31+ cells (green fluorescent by IHC), rabbit anti-phospho-γH2AX (Ser139) primary antibody (1:100, Cell Signaling Technology, Danvers, MA) was visualized in red using a secondary goat anti-rabbit IgG Alexa Fluor 594 (1:200, Abcam). Cells were visualized and photographed as described below.

### Apoptosis (TUNEL staining)

To detect later stages of cell apoptosis, the terminal deoxynucleotidyl transferase dUTP Nick End Labeling (TUNEL) assay was used to identify cells undergoing DNA fragmentation as a result of DNA damage. The TUNEL assay was performed according to standard protocols [62] in combination with fluorescent immunostaining to identify CD31+ endothelial cells and desmin+ myogenic cells. TUNEL staining was always performed separately from the proliferation assay using BrdU incorporation [63].

Coverslips were fixed and incubated with 0.2% Triton X-100 in PBS-Tween 20 (PBS-T) for 30 mins, and washed with PBS-T. Coverslips were incubated with 3% peroxide (Sigma-Aldrich) in PBS for 10 min, and washed with PBS-T and double-distilled water. Coverslips were incubated with terminal deoxynucleotidyl transferase (ThermoScientific, Burlington, ON, Canada) reaction buffer (Sigma-Aldrich) for 30 mins at room temperature (Epicentre, Madison, WI), before overnight incubation with primary antibodies (anti-CD31, anti-desmin) to detect cell type, together with anti-BrdU antibody (to detect nick-end labeling of DNA fragments by BrdUTP). Coverslips were washed in Tris-buffered saline containing Tween20 (TBS-T), and incubated for 2 hr in the dark at room temperature in a solution containing the appropriate secondary antibodies to detect endothelial and myogenic cells (as described, above) and washed again in TBS-T. To visualize apoptotic cells, coverslips were incubated with DAB solution. After this incubation, the coverslips were washed multiple times with ddH_2_O and mounted on clean glass slides using Vectashield (Vector Laboratories, Inc., Burlingame, CA). The apoptotic events for each cell type were visualized and photographed as described below.

### Inhibitory immunoneutralization

To test whether the effects on proliferation and early- and late-stage apoptosis were induced by the treatments, corresponding semaphorin antibodies were applied to neutralize each treatment. A 40-fold concentration of each antibody relative to the molar equivalent of the respective semaphorin (anti-Sema3A, AB9604, Merck Millipore, ON, Canada; anti-Sema3F, ab39955, Abcam) was applied to the corresponding treatment groups. The two anti-semaphorin antibodies are used for epitope detection in western blots and tissue sections, and have not previously been shown to neutralize effects of semaphorins. Immunostaining was used to identify endothelial and myogenic cells for counting.

### Imaging and analysis

Cells stained with one or more antibodies were observed using a Zeiss Apotome microscope (Zeiss Canada, Toronto, ON), and photographed at 200x magnification from 8 non-overlapping fields per coverslip. Image files were converted to TIF format using Zeiss Zen software. Multi-color images were analyzed by assessing each cell for staining for CD31 (green), desmin (red), and/or BrdU (dark staining in bright-field images). DAPI counterstaining (blue) was used to identify cell nuclei. Staining for γH2AX marked DNA damage and TUNEL staining was used to detect apoptotic events in 8 non-overlapping fields imaged from each coverslip. Cells were counted using NIH ImageJ 1.50i software. Cell counts or proportions for each image were averaged for the 8 non-overlapping fields imaged from each coverslip; data for each coverslip were compiled by treatment group in a Microsoft Excel 2016 spreadsheet for analysis and graphing. The mean ± standard error of the mean (SEM) used to represent each treatment in bar graphs was calculated as the mean of the averages for all cultures in that group. Data were expressed as the number of surviving cells per field (density) or the fraction cells that were either: CD31+ and desmin+ cells stained positive for BrdU (DNA synthesis), γHA2X (DNA damage), TUNEL+ (late apoptosis), or the ratio of BrdU+ endothelial cells to BrdU+ myoblasts.

Statistical differences in cell number and proportions among treatment groups were analyzed with analysis of variance (ANOVA) and *post hoc* Tukey's means tests. A probability of p<0.05 was used to indicate statistical significance.

## Abbreviations

αA, αF or αA+αF (in figures): neutralizing antibody to Sema3A Sema3F, or both
A, F or A+F (in figures): semaphorin 3A, 3F, and 3A+3F
ANOVA: analysis of variance
BrdU: bromodeoxyuridine
CD31: cluster of differentiation 31
DAB: 3,3'-diamino-benzidine
DAPI: 4,6-diamidino-2-phenylindole
H2AX: histone 2 AX
HGF: hepatocyte growth factor
IHC: immunohistochemistry
mTOR: mammalian target of rapamycin
PBS-T: phosphate buffered saline with Tween
SEM: standard error of the mean
Sema3A: semaphorin 3A
Sema3F: semaphorin 3F
Sema3A+3F: semaphorins 3A and 3F, combined
TBS-T: Tris-buffered saline with Tween-20
TUNEL: terminal deoxynucleotidyl transferase dUTP Nick End Labeling
